# Computational Analysis of the Behavior of BODIPY Decorated Monofunctional Platinum(II) Complexes in the Dark and under Light Irradiation

**DOI:** 10.1021/acs.jpca.2c04544

**Published:** 2022-10-04

**Authors:** Pierraffaele Barretta, Fortuna Ponte, Stefano Scoditti, Vincenzo Vigna, Gloria Mazzone, Emilia Sicilia

**Affiliations:** † Department of Chemistry and Chemical Technologies, University of Calabria, Ponte P. Bucci, 87036 Arcavacata di Rende (CS), Italy

## Abstract

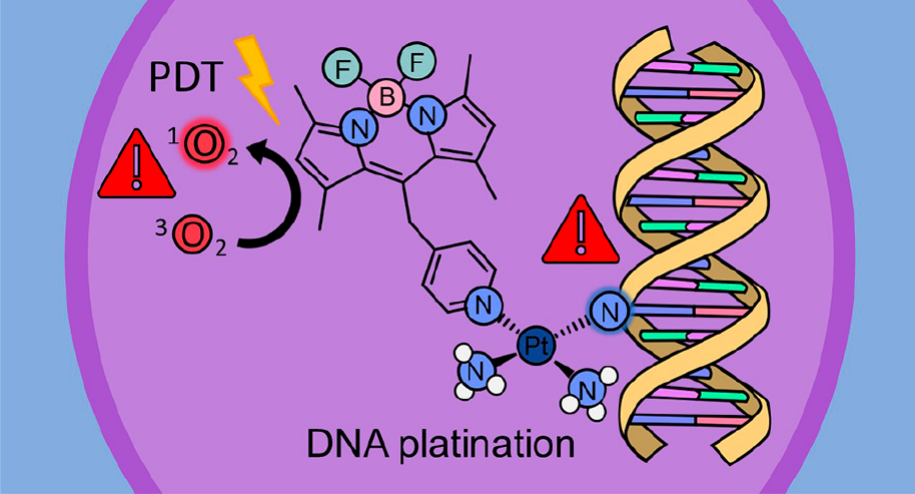

Dual-action drugs are occupying an important place in the scientific landscape of cancer research owing to the possibility to combine different therapeutic strategies into a single molecule. In the present work, the behavior of two BODIPY-appended monofunctional Pt(II) complexes, one mononuclear and one binuclear, recently synthesized and tested for their cytotoxicity have been explored both in the dark and under light irradiation. Quantum mechanical DFT calculations have been used to carry out the exploration of the key steps, aquation and guanine attack, of the mechanism of action of Pt(II) complexes in the dark. Due to the presence of the BODIPY chromophore and the potential capability of the two investigated complexes to work as photosensitizers in PDT, time dependent DFT has been employed to calculate their photophysical properties and to inspect how the sensitizing properties of BODIPY are affected by the presence of the platinum “heavy atom”. Furthermore, also the eventual influence on of the photophysical properties due to the displacement of chlorido ligands by water and of water by guanine has been taken into consideration.

## Introduction

Despite the great effort dedicated to the search of new and more active metal containing antineoplastic agents, cisplatin and its Pt(II) derivatives remain the most effective drugs in inhibiting proliferation of cancer cells.^1−4^ However, as their clinical utility is strongly limited by severe side effects, tumor resistance, and undesired normal tissue toxicities,^5−7^ several structural modifications to both N-donor nonleaving and leaving ligands of cisplatin have been made in the attempt to circumvent such well-known drawbacks. Among the strategies pursued to improve the therapeutic index and decrease side effects, new classes of platinum compounds such as monofunctional platinum(II) complexes showing antineoplastic activity have been proposed as nonclassical alternatives.^8−10^ Similar to cisplatin and its derivatives, monofunctional platinum drugs undergo aquation inside the cell followed by nuclear DNA binding, which are the key activation steps of platinum-based drugs. Unlike cisplatin, nonetheless, only a single bond can be formed with DNA, as in such drugs only one labile ligand is bound to the metal center, and due to additional interactions and steric hindrance, cellular repair ability via transcription is reduced and apoptosis is induced with a different mechanism.^11,12^ Aiming at enhancing the efficacy of monofunctional anticancer agents, dual-action complexes have been designed by appending a suitable photosensitizer (PS) to the ligand bound to platinum(II). Two-component systems of this kind have recently received increasing attention due to the possibility to combine the DNA cross-linking ability of the complex bearing a labile anionic ligand, generally a chloride, with the photodynamic therapy (PDT) effect of a PS.^13−18^ PDT is an alternative treatment for the control of malignant diseases based on the uptake of a photosensitizing molecule which, upon being excited by light of proper wavelength, reacts with oxygen and generates oxidant species in target tissues, leading to cell death.^19,20^ The PS, in the photodynamic process, is promoted from its ground singlet state to an excited one. The excitation is followed by an intersystem crossing (ISC) transition to a lower triplet state, being a usually forbidden nonradiative process that can take place if the relativistic spin–orbit coupling (SOC) between the two states is large enough and the involved states are close in energy to permit the process to be efficient.

Boron dipyrromethanes (BODIPYs) are fluorescent dyes that have attracted considerable attention due to their excellent photochemical properties^21−23^ and have been widely used in combination with Pt(II) chemotherapeutic agents also because the introduction of the Pt atom in the BODIPYs structure should increase their efficacy in generating singlet oxygen enhancing the intersystem crossing, thereby favoring spin–orbit coupling.^17,24−26^

**Scheme 1:**
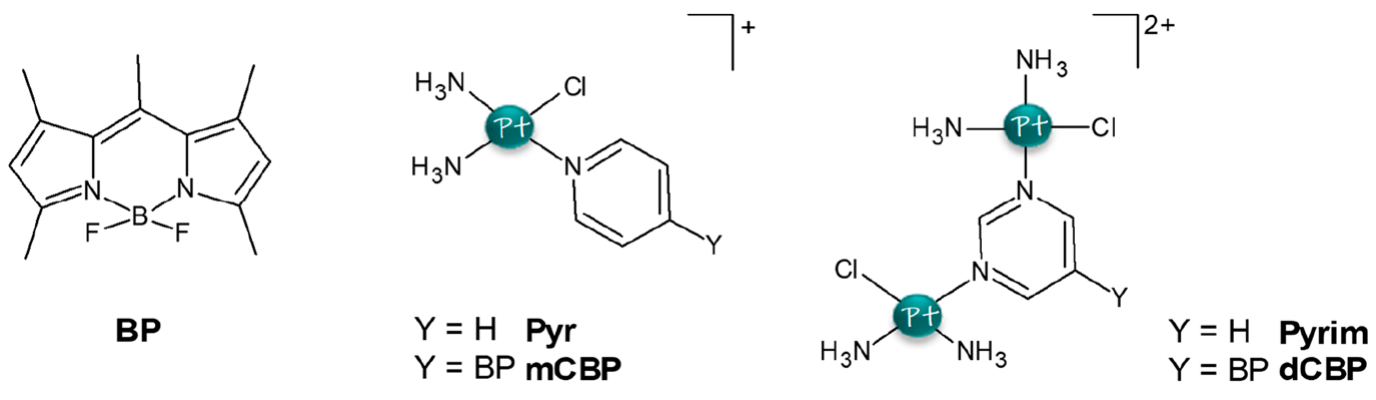
Schematic Representation of the Investigated Compounds: the BODIPY Chromophore (**BP**), the Pyridine- (**Pyr**) and Pyrimidine-Chelated (**Pyrim**) Cisplatin Complexes, and the Two BODIPY-Appended Pt(II) Complexes **mCBP** and **dCBP**

Given such premises and due to the prominent place that dual-action drugs are occupying in the scientific landscape of cancer research thanks to the possibility to combine different therapeutic strategies within one molecule, we have carefully examined both the photophysical properties and the key steps of the mechanism of action of two BODIPY-appended monofunctional Pt(II) complexes recently synthesized and tested for their cytotoxicity.^16,18^ The structures of the two investigated complexes named in the original papers **mCBP** {[*cis*-Pt(NH_3_)_2_Cl]-8-(*p*-pyridinemethylene),1,3,5,7-tetramethyldipyrrinborondifluoride}^+^ and **dCBP** {[*cis*-Pt(NH_3_)_2_Cl]_2_-8-(1,3-pyrimidine-5-methylene),1,3,5,7-tetramethyldipyrrinborondifluoride}^2+^, are shown in Scheme 1, with the latter being a binuclear Pt(II) complex. For both compounds, it is reported that they show promising ROS generating capability under light irradiation reinforcing the classical chemo-therapic action in the dark caused by DNA distortion. In addition, it has been hypothesized that the binuclear **dCBP** complex is able to cause enhanced DNA damage due to its DNA cross-linking capability.^16^

Quantum mechanical DFT and time dependent DFT calculations have been used to carry out a detailed investigation of some aspects of the cytotoxic activity of such complexes by examining the key steps of the mechanism of action of Pt(II)-based drugs and photophysical properties and how such properties are influenced by the substitution of the chlorido ligand with water and by the interactions established with DNA bases.

## Computational Details

Quantum mechanical DFT calculations have been performed using the Gaussian16 code.^27^ The hybrid Becke three parameter exchange functional^28^ and the Lee–Yang–Parr correlation functional^29^ (B3LYP) have been employed, including the Grimme dispersion corrections for nonbonding interactions through an atom pairwise additive scheme, DFT-D3,^30^ for the full geometry optimizations of all the minima and transition states. Optimizations have been carried out in implicit water (ε = 78.4) using the PCM continuum solvation model as implemented in Gaussian 16.^31,32^ The SDD effective core potential^33^ and the corresponding valence basis set have been used to describe platinum atoms. The 6-31G(d,p) basis set has been employed to describe the rest of the atoms. Frequency calculations have been performed at the same level of theory for both, confirming the nature of minima and transition states of stationary points located along the reaction pathways and including zero-point energy correction calculations.

Aiming at accurately describe the photophysical properties of the systems under investigation, a preliminary benchmark study has been carried out on the maximum absorption wavelength of both **BP** and **mCBP**. For this purpose, TDDFT calculations on the B3LYP-D3-optimized structures have been performed using a series of exchange-correlation functionals. Specifically, the performance of the GGA (general gradient approximation) functionals B97D^34^ and PBE0,^35^ the meta-NGA (nonseparable gradient approximations) MN12L^36^ and MN15L,^37^ the meta-GGA M06L,^38^ the global-hybrid GGA B3LYP, PBE0, and B3PW91,^39^ the global-hybrid meta-GGA M06,^40^ and the range-separated hybrid GGA cam-B3LYP^41^ and ωB97X^42^ have been tested. Though the available experimental spectrum of **BP** has been recorded in acetonitrile, a very similar behavior in aqueous environment has been previously found.^43,44^ Accordingly, all the TDDFT calculations have been performed in water by employing the same implicit solvent model used for the optimizations. TDDFT outcomes have been compared also with the excitation energies computed at the spin-component scaling second-order approximate coupled-cluster (SCS-CC2) level, suggested to be a good approach, even if high time-demanding, in reproducing the excitation energies of several boron-containing chromophores.^45−47^ SCS-CC2 calculations have been computed with the aid of the Turbomole package,^48,49^ employing the def2-SVP basis sets for all the atoms and the corresponding effective core potential for the platinum center.^50^ All the obtained data have been collected in Tables S1.

In order to ascertain that an intersystem spin crossing from a bright singlet state to a triplet one can occur, spin–orbit matrix elements have been computed using the SOC-TD-DFT approach, as implemented in the Orca package,^51^ employing the ωB97X functional. Relativistic corrections have been computed by the zeroth order regular approximation (ZORA) at the ground state optimized geometries. Accordingly, ZORA-DEF2-SVP and SARC-ZORA-SVP basis sets for the main and metal atoms, respectively, have been employed and the SOCs values calculated as previously reported.^52^

## Results and Discussion

### Aquation and Guanine Binding Reactions of mCBP and dCBP Complexes

**Figure 1:**
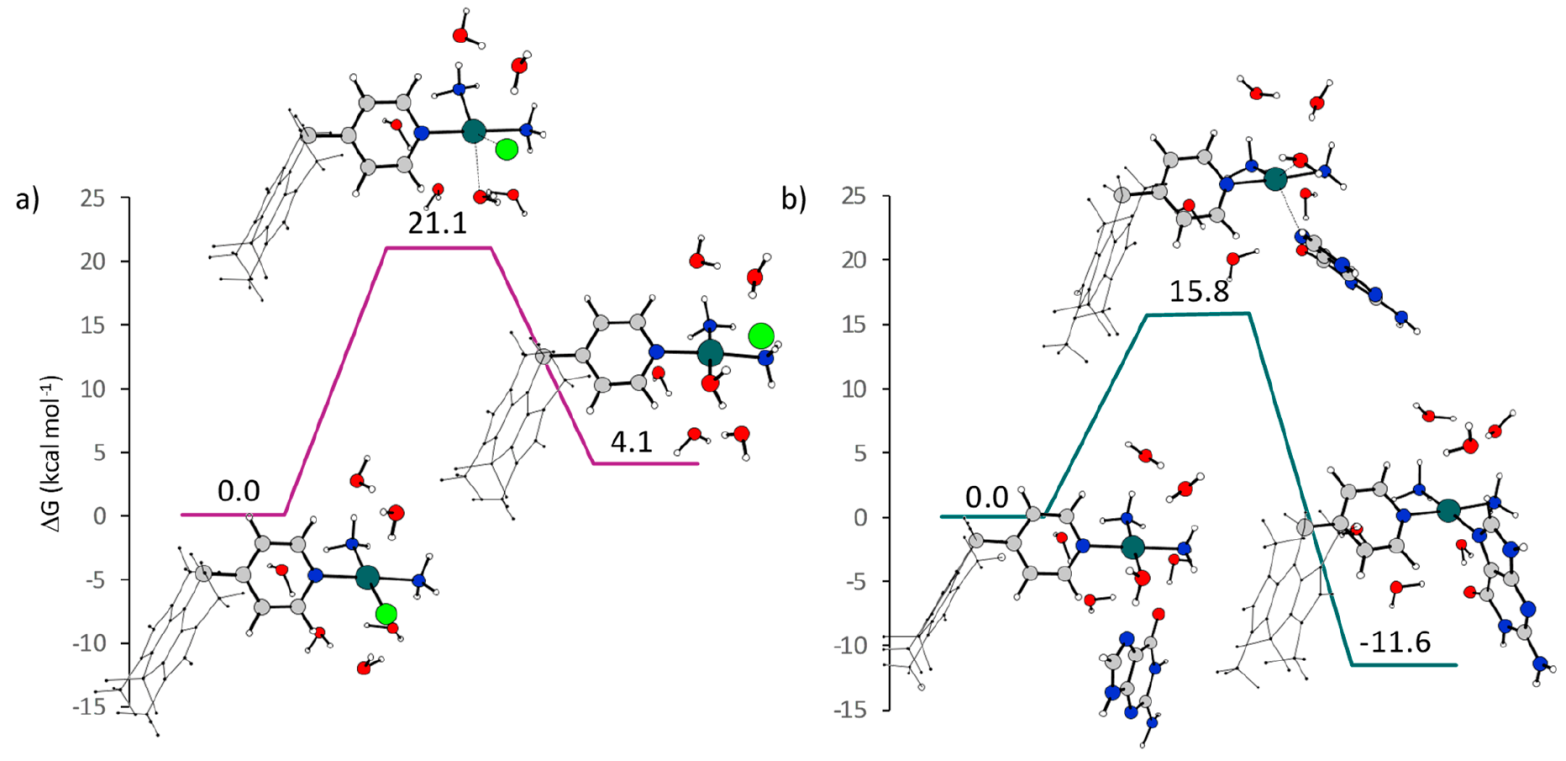
Free energy profiles in water describing the aquation and guanine interaction of the **mCBP** complex. Relative free energies are in kcal mol^–1^ and have been calculated with respect to the first formed adduct.

The mechanism of action of monofunctional platinum drugs, in analogy to cisplatin and its derivatives, involves aquation, that is promoted inside the cell where chlorido concentration is much lower than outside, followed by DNA binding. These two well-known activation steps of platinum-based drugs have been computationally examined for both **mCBP** and **dCBP** complexes and compared with the analogous behaviors of cisplatin and parent pyriplatin (**Pyr**) adopting the same computational approach described above. The outcomes of our analysis for **mCBP** and **dCBP** are collected in Figures 1 and S1 and Table 1. Figure 1 reports the free energy profiles calculated in water solvent for the **mCBP** complex together with a sketch of the intercepted stationary point geometrical structures.

**Table 1:** Calculated Values of the Activation Free Energies (Δ*G*^‡^ in kcal mol^–1^) and Reaction Free Energies (Δ*G*_react_ in kcal mol^–1^) for the Hydrolysis and Guanine Attack Reactions of **mCBP** and **dCBP** Complexes, Compared with Those of Cisplatin, **Pyr**, and **Pyrim**

compound	process	**Δ***G*^**‡**^	**Δ***G*_**react**_
**mCBP**	hydrolysis	21.1	4.1
	guanine attack	15.8	–11.6
**Pyr**	hydrolysis	21.1	3.7
	guanine attack	18.3	–9.8
cisplatin	hydrolysis	21.6,^a^ 23.3	3.6,^b^ 4.2,^c^ 5.2
	guanine attack	18.0	–10.4
**dCBP**	first hydrolysis	19.3	0.8
	second hydrolysis	20.7	4.8
	first guanine attack	20.9	–10.4
	second guanine attack	22.0	1.0
**Pyrim**	first guanine attack	21.1	–7.0
	second guanine attack	21.9	3.8

a Mean value of data reported in refs (53−59).

b Reference (54).

c Reference (53).

A second-order nucleophilic substitution (S_N_2) reaction allows the substitution of the chlorido anion with a water molecule. The interaction between the reacting species leads to the formation of a first adduct to which, in order to better reproduce the solvent environment, five explicit water molecules have been added. The transition state for the associative displacement of the chlorido ligand, possessing a pseudo trigonal bipyramidal geometrical structure, lies 21.1 kcal mol^–1^ above the zero reference energy of the first formed adduct (see Figure 1a). Formation of the aquated product, named **mCBP**_**w**_, is calculated to be endergonic by 4.1 kcal mol^–1^. Therefore, the barrier height falls in the range of the values previously calculated and experimentally estimated for cisplatin^53−59^ that goes from 19 to 24.1 kcal mol^–1^ for an average value of 21.6 kcal mol^–1^, as well as the destabilization of the product with respect to the initial adduct is perfectly superimposable with the cisplatin estimated experimental values (3.6 and 4.2 kcal mol^–1^). The corresponding calculated values for cisplatin, obtained adopting the same computational protocol, are 23.3 and 5.2 kcal mol^–1^ for the energy barrier and reaction energy, respectively. Details can be found in Figure S2 of the Supporting Information. The value of the barrier height calculated for **Pyr** is 21.3 kcal mol^–1^, whereas the product is calculated to be destabilized by 3.7 kcal mol^–1^. Both values are comparable to those of the **mCBP** complex.

The outcomes of our computational analysis of the interaction of the **mCBP** complex with DNA, simulated considering a guanine nucleobase as a model, are illustrated in Figure 1b. The attack at the N7 position of guanine causing the displacement of the water ligand takes place by the formation of an initial adduct between the reacting species stabilized by the formation of hydrogen bonds between both the guanine N7 and the leaving water molecule and the oxygen of guanine with one of the ammonia ligands. The interacting species properly oriented one with respect to the other reaction, overcoming a barrier for the associative displacement of 15.8 kcal mol^–1^ and leading to the coordination of the guanine base that results to be exergonic by 11.6 kcal mol^–1^ with respect to the zero reference energy of the initial adduct. The reaction product has been named **mCBP**_**g**_. For the same substitution to occur in cisplatin, we have calculated a barrier of 18.0 kcal mol^–1^ and a product stabilization of 10.4 kcal mol^–1^ with respect to the initial cisplatin guanine adduct. Analogous calculations carried out to describe the water/guanine exchange in **Pyr** give 18.3 kcal mol^–1^ for the barrier and −9.8 kcal mol^–1^ for the reaction energy. Free energy profiles in water are reported in Figure S3 of the Supporting Information. In order to make the comparison easier, the barrier height and reaction energy values for both aquation and guanine attack have been collected in Table 1.

The values of the barriers and the energetics calculated for the analogous aquation and guanine platination reactions of the complex **dCBP** are also collected in Table 1, whereas the corresponding free energy profiles and the geometrical structures of the intercepted stationary points are depicted in Figure S1. The **dCBP** complex is activated by the first aquation involving the chlorido ligand of one of the Pt(II) units. The pseudo bipyramidal geometrical structure of the transition state lies 19.3 kcal mol^–1^ above the energy of the initially formed adduct, whereas the product of the chlorido displacement is destabilized by only 0.8 kcal mol^–1^ with respect to it. The second hydrolysis takes place with the displacement of the second chlorido ligand by the incoming water molecule, with the activation and the reaction free energy being 20.7 and 4.8 kcal mol^–1^, respectively. The product of the double aquation has been indexed as **dCBP**_**2w**_.

The DNA interaction has been simulated by the formation of two new covalent bonds with the model guanine base at the N7 position displacing the water ligands of the diaquated **dCBP** complex **dCBP**_**2w**_. The transition state for the first associative displacement lies 20.9 kcal mol^–1^ above the energy of the adduct stabilized by the formation of several hydrogen bonds established with the incoming guanine molecule. The reaction leading to the formation of the **dCBP**_**wg**_ product results to be exergonic by 10.4 kcal mol^–1^. The second attack proceeds by overcoming an energy barrier of 22.0 kcal mol^–1^ calculated with respect to the entrance channel, and the whole reaction for the formation of the complex bound to two guanines, termed **dCBP**_**2g**_, is endergonic by 1.0 kcal mol^–1^.

On the basis of such results, it appears that the behavior of the binuclear complex is very similar to that of cisplatin, **Pyr** and **mCBP** when the aquation reactions are taken into consideration. The DNA platination, instead, simulated through the attack of the guanine nucleobase, shows significant differences with respect to the average behavior. Indeed, both barriers for the first and second attacks are higher than those calculated for cisplatin, **Pyr**, and **mCBP**, and very importantly, the reaction energy for the second attack is even slightly endothermic.

This behavior not in line with the general trend is, very likely, due to the steric constraints imposed by the structure of the complex that does not favor the approach between the interacting species, in particular when the second attack occurs due to the contemporary presence of two guanine molecules (see Figure S1). For the sake of comparison and for checking whether the BODIPY moiety influences the properties of the investigated complex, the two sequential water displacements by guanine in the simple pyrimidine-chelated cisplatin complex (**Pyrim**) also have been explored, as reported in Figure S4. When the guanine interaction with the aquated **Pyrim** complex is examined, the heights of the barriers are 21.1 and 21.9 kcal mol^–1^ and the reaction energies are −7.0 and 3.8 kcal mol^–1^ for the first and second attack, respectively. From a comparison between the values obtained for **dCBP** and its parent **Pyrim**, it appears that the presence of the appended BODIPY does not play a significant role at this stage and the more sterically hindered structure of the binuclear Pt complex, regardless the presence of the BODIPY moiety, penalizes the second guanine attack. According to the enhanced experimentally observed loss of DNA helical structure for **dCBP**,^16,18^ it can be inferred from such results that, in analogy to phenanthriplatin,^60,61^ the first attack to a guanine base causes an initial DNA double helix distortion. Such a distortion could lead to a rearrangement of the real DNA flexible structure more favorable to the second guanine attack and, therefore, to cross-linking.

Summarizing the results reported above and collected in Table 1, it appears that the hydrolysis reaction of both **mCBP** and **dCBP** does not present any anomalous behavior when compared with that of the reference compound cisplatin. Guanine attack, which simulates the first step of the DNA platination, is in line with that of cisplatin when the mononuclear **mCBP** complex is examined, whereas the double water displacement by guanine process is calculated to be less favorable for the binuclear **dCBP** complex, very likely as a consequence of the sterically hindered structure of the complex.

### Photophysical Properties

As it has been previously reported, the TD-DFT approach often overestimates the low-lying excited states of BODIPY-containing systems,^62,63^ by even more than 0.3 eV. Several efforts have been devoted to a better reproduction of the spectral features of BODIPYs.^64,65^ Nonetheless, due to the rather systematic nature of this deviation, TDDFT usually works well in predicting the shifts in energy induced by the introduction of various substituents or by chemical modifications of the BODIPY core. Thus, in order to properly select the most appropriate functional for the exploration of the photophysical properties of **BP**, **mCBP**, **dCBP**, and all the plausible derivatives obtained by the activation in the cellular environment, which are aquated and guanine-bound complexes, a preliminary investigation has been carried out on the performance of a series of exchange-correlation functionals in reproducing the most relevant absorption peak in PDT, the maximum absorption wavelength. For this purpose, TDDFT on the B3LYP-D3 optimized structures of both **BP** and **mCBP** have been carried out employing various kinds of functionals (see Table S1). The obtained data have been compared also with the outcomes of the more accurate approach, SCS-CC2. As expected, TDDFT results evidence that all the functionals systematically overestimate the first excitation energy, from ∼0.4 up to ∼0.6 eV found for both **BP** and **mCBP** when meta-NGA functionals are used. The functionals that are able to better reproduce the recorded maximum absorption wavelength are M06, B97D, ωB97X, and PBE. However, on the basis of the observed efficacy of range-separated hybrid functionals in reproducing the fundamental gap and charge transfer state energies of molecular systems in the condensed phase,^66^ ωB97X has been selected and adopted for all of the other TDDFT calculations. Moreover, it is worthy of note that even the more accurate SCS-CC2 approach return a maximum absorption wavelength for the two systems with an error of 0.3 eV; therefore, the chosen ωB97X provides as much accuracy as a possible for the reproduction of one of the most important quantities in PDT with a considerable reduction of the computational cost.

#### Calculated Electronic Spectra for **mCBP** and **dCBP** Complexes and Their Aquated and Guanine Bound Derivatives

**Figure 2:**
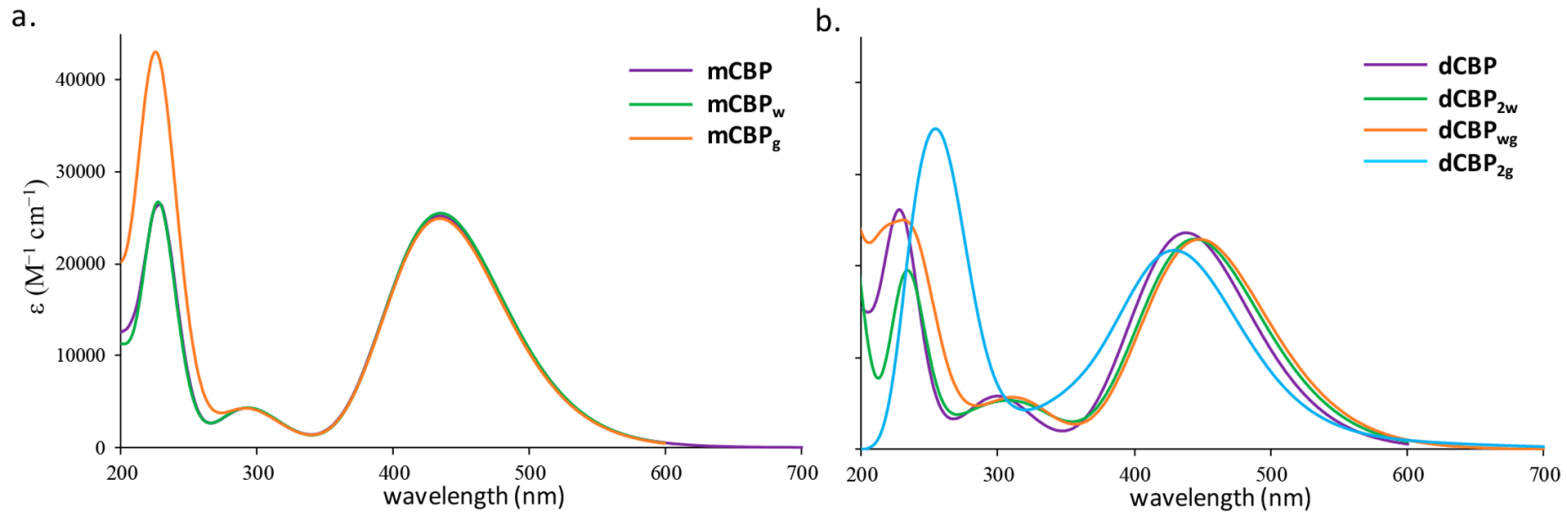
Computed absorption spectra of the (a) intact **mCBP** complex (violet) and its aquated (green) and guanine bound (orange) forms and (b) intact **dCBP** complex (violet) and its aquated (green) and one (orange) and two guanine bound (light blue) forms.

Vertical excitation energies, maximum adsorption wavelength, oscillator strength and molecular orbitals (MO) contributions for singlet excitations are collected in Table S2 of the Supporting Information and compared with the available experimental data.^16,67^ Calculated spectra of **mCBP** is illustrated in Figure 2a. In order to simplify the description of the nature of the MOs participating in the calculated transitions as occurring between an excited particle and an empty hole, natural transition orbitals, NTOs, are also provided in Figure S5 of the Supporting Information for both singlet lowest-energy transitions. Lowest lying triplet excitation energies together with MO contributions are reported in Table S3, and NTOs are included in Figure S5.

Since the photodynamic action involves the activation of the PS by irradiation that leads to the population of the low-lying singlet state and the subsequent ISC process through an energy transfer to a triplet state lying below, only triplet states with energies lower than that of the bright singlet one have been taken into consideration. As previously anticipated, the changes in the spectra, as well as in other photophysical properties, of the **mCBP** and **dCBP** complexes induced by the displacement of the chlorido ligand by water and of water by guanine have been explored. The calculated electronic spectra are reported in the same Figure 2, whereas all the information concerning singlet and triplet transitions are collected in Tables S2 and S3. The number of triplet states taken into account depends on the considered species as evidenced in Table S3. Also for **mCBP**_**w**_, **mCBP**_**g**_, **dCBP**_**2w**_, **dCBP**_**wg**_, and **dCBP**_**2g**_ NTOs have calculated and plotted in Figures S5–S7.

The experimentally detected absorption spectra in solution, which are acetonitrile and PBS for **BP**(67) and Pt(II) complexes,^16^ respectively, display essentially a band centered at around 500 nm. The wavelength of maximum absorbance in the experimental recorded absorption spectrum of **BP** is 491 nm (see Table S2) and the corresponding λ_max_ values for the two **mCBP** and **dCBP** complexes are 503 and 506 nm, respectively. The corresponding values in the computed spectra are 423 nm for **BP**, 434 nm for **mCBP** and 438 nm for **dCBP**. Therefore, in agreement with experiments, the absorption spectra in the range 400–700 nm of the two Pt(II) complexes remain almost unchanged with respect to that of the parent BODIPY indicating that the platinum introduction does not affect the photophysical properties of the appended dye. Such absorption bands are originated by HOMO → LUMO transition in both cases, and according to the central role played by the dye, the characteristic sharp and intense BODIPY lowest energy spin-allowed transition of ππ* character assumes ILCT (intraligand charge transfer) character when the Pt(II) complexes are examined as reported in Table S2. As it clearly appears from NTO plots the charge transfer that accompanies the formation of the excited states involves only the BODIPY moiety (see Figure S5).

The spectra calculated for **mCBP** both in its aquated form, **mCBP**_**w**_, and bound to a guanine free molecule as a DNA model, **mCBP**_**g**_, by displacement of water are reported and compared with that of the intact complex in Figure 2. From such a figure and data reported in Table S2, it is readily apparent that the presence neither of water nor of guanine causes any change in the spectral features, especially in the low-energy region, the most important in PDT. Two different bands can be distinguished: the first one always originated by the same transition as in the intact complex **mCBP** and with the same ILCT character; the second band, instead, is determined by a charge transfer from the platinum to the bipy ligand (MLCT) in all cases, although the intensity of the band differs on the basis of the considered species.

The situation is more diversified when the substituted derivatives of **dCBP** are examined. Indeed, a general red-shifting of the wavelengths, though of only a few nanometers, is observed upon aquation (**dCBP**_**2w**_) and modeled partial DNA platination (**dCBP**_**wg**_), while a slight blue-shift occurs with the complete substitution of water ligands with guanine ones (**dCBP**_**2g**_). The electronic transition occurs from the HOMO (H) to the LUMO (L) in the former cases and becomes H → L + 1 in the latter one. However, as showed by the NTOs reported in Figure S6 it remains always centered on the BP ligand (ILCT) as in the case of mononuclear species and is essentially characterized by the same intensity. The DNA platination simulated for the **dCBP**_**2g**_ complex provokes the appearance of the H → L transition at 588 nm with an oscillator strength of 0.016, which could cause the drug to be activated once it reaches its main target by a deep penetration of the radiation. The NTO contour plots of the first singlet state show that both the metal centers are involved together with the pyrimidine, dictating a contribution of LMCT character to the main absorption band absent in the mononuclear species.

#### Properties of Excited States

**Figure 3:**
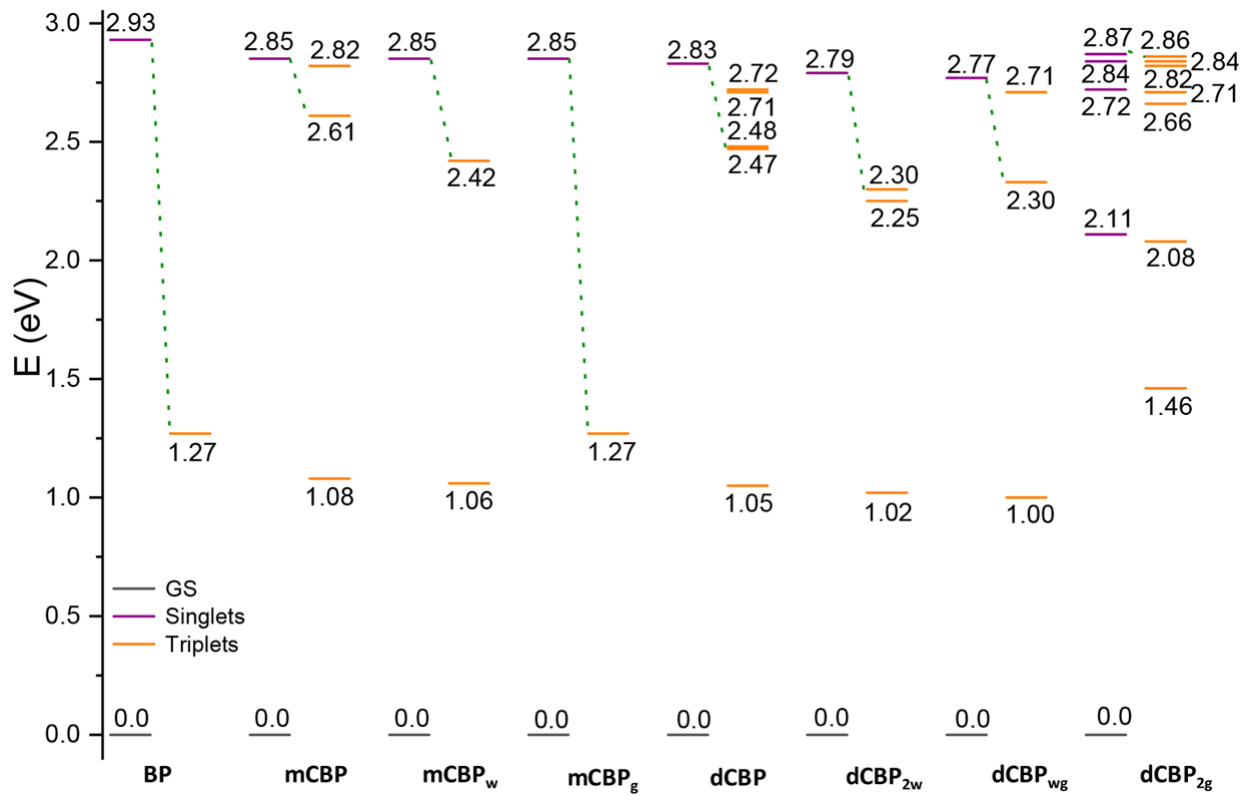
Energy diagram of the low-lying excited singlet (purple) and triplet (orange) states of **BP**, **mCBP**, **dCBP,** and all their water- and guanine-derivatives computed with respect to the ground (black) state zero energy. Dotted green lines indicate the most probable ISC channel.

It is well established that the efficiency of compounds proposed as photosensitizers in PDT depends on the energies of the low-lying triplet states that have to be not smaller than the threshold value of the energy gap between the triplet molecular oxygen ground state (^3^O_2_) and its singlet excited state (^1^O_2_), which is 0.98 eV. Energy gaps between the ground-states and the first triplet excited states, as well as all the states potentially involved in the entire PDT process, for all the compounds examined here, are depicted in Figure 3 and described in detail in Table S3. From such values it appears that all the calculated T1 energies are larger than the 0.98 eV threshold. Relative energy slightly decreases with the displacement of the chlorido ligand in favor of water (**mCBP**_**w**_ and **dCBP**_**2w**_) and considerably increases when guanine enters the coordination sphere of the metal in place of water ligands (**mCBP**_**g**_ and **dCBP**_**2g**_). The spin-forbidden triplet transition at lowest-energy of **mCBP** and its derivatives is due to the H → L transition and, therefore, possess ILCT character. The same is for **dCBP** and its derivatives formed by displacement of one chloride and one water, while for the **dCBP**_**2g**_ it is originated by a H → L + 1 transition. In all cases, such triplet states are centered on the BP ligand and then possess ILCT character.

The PDT applicability of potential PSs relies on the ISC probability, which provides more time for the triplet excited state to interact with dioxygen. Then, spin–orbit coupling matrix elements, reflecting the ISC probability, have been calculated and gathered in Table S4, together with the singlet–triplet splitting energies potentially involved in the process. On the basis of such values, the most probable couplings are shown with the green dotted lines in Figure 3. The values of the SOC elements calculated for the coupling of the bright excited singlet state of the free **BP** with the triplet state T1 lying below is 0.05 cm^–1^. For the mononuclear complex **mCBP** there are three excited triplet states with different character lying below the first excited singlet one. While, as stated above, T1 mainly involves the **BP** core, in T2 and T3 a considerable participation of the metal in both hole and particle plots can be observed, determining a mixed MC/MLCT character of such states.

The substitution of chloride with water, for activating the complex from a chemotherapy point of view, and the subsequent exit of water in favor of DNA binding, here simulated with a guanine base, entail a gradual decrease of the triplet states number with lower energy than the bright singlet one. Among the two triplet states found for **mCBP**_**w**_, only T2 evidences a contribution of the metal to the NTOs isodensity. On the other hand, the presence of guanine bound to the metal center in **mCBP**_**g**_ maintains the triplet state entirely centered on the **BP** ligand, and neither the metal nor the pyrimidine portion is involved in the CT. The different number of excited triplet states with energy lower than the bright singlet one implies that, depending on the involved species, intact **mCBP**, aquated **mCBP**_**w**_, or guanine-bound **mCBP**_**g**_, different S1 deactivation channels are accessible. Although almost all the SOCs computed for **mCBP** and its derivatives are larger than those for the free **BP**, so the presence of the Pt center does not influence the coupling so much, being the SOC values comprised between 0.02 and 2.54 cm^–1^. However, as stated above, in all the species of the mononuclear complex, **mCBP**, the main absorption band, associated with the S1 excited state, is centered on the **BP** ligand, and thus, it is of the ILCT type (see Figure S5). Accordingly, the largest couplings have been calculated for triplet states with different character and energy closest to the bright one (e.g., the S1-T2 for **mCBP**).

In analogy with **mCBP**, for **dCBP**, spin–orbit coupling is also more efficient than for **BP** alone, and the platinum-induced heavy atom effect seems to cause a more significant enhancement of the SOC values, especially for the complex anchored to the DNA bases (**dCBP**_**2g**_). While, for the **dCBP** complex and its aquated and monoguanine bound derivatives, the coupling, very likely, occurs among S1 (ILCT) and T2 states with mixed MC/MLCT character (Figure S7), for the **dCBP**_**2g**_ complex the brightness of the fourth singlet state gives access to several deactivation pathways that could in principle involve not only the bright state but also other singlet states lying below it that can be populated by internal conversion (IC). Indeed, the coupling of both S2 and S3 with the triplet states lying below abundantly exceeds the value of 15 cm^–1^ and comes to be more than 70 cm^–1^ for the S3-T6. Nevertheless, the largest value computed for such a complex involves the bright singlet state and the triplet state closest in energy (T7). The radiationless transition starts from a ILCT state and ends in a mixed MC/MLCT state. Thus, it is reasonable to hypothesize that the S4-T7 coupling could be the most probable deactivation channel.

## Conclusions

DFT and TDDFT have been employed to carry out an investigation of some aspects of the cytotoxic activity of two photosensitizing monofunctional Pt(II) complexes, one mononuclear (**mCBP**) and one binuclear (**dCBP**), decorated with a BODIPY. Free energy profiles in water describing the classical steps, that is aquation and DNA interaction simulated by the attack to a guanine molecule as a model, of the mechanism of action of Pt(II) drugs have been calculated. The comparison with the cisplatin complex and parent pyriplatin shows that, along the hydrolysis paths, both **mCBP** and **dCBP** follow a typical trend being the energy barriers in the range between 19.0 and 21.0 kcal mol^–1^ and the whole reaction is endothermic. The description of the DNA interaction, simulated through the attack on the N7 position of a free guanine, is in line with the general trend for **mCBP** as the height of the energy barrier is 15.8 kcal mol^–1^ and the whole reaction is exothermic by 11.6 kcal mol^–1^. The behavior is different for the binuclear **dCBP** as both barriers for the first and second attacks are higher than those calculated for cisplatin, pyriplatin and **mCBP**, and very importantly, the reaction energy for the second attack is even slightly endothermic. The more sterically hindered structure of the binuclear Pt complex, very likely, with the first attack causes an important DNA structure distortion that might lead to an arrangement of the two species more favorable to the second guanine attack.

As a consequence of the presence of the BODIPY chromophore and the potential capability of the two investigated complexes to work as photosensitizers in PDT, time dependent DFT has been employed to calculate their photophysical properties and to inspect how the sensitizing properties of BODIPY are affected by the presence of the platinum “heavy atom”. The outcomes of our calculations show that the spectra of the complexes are very similar to that of the free BODIPY and that the substitution of the chlorides by water and of water by guanine causes a slight red shifting of the λ_max_ only for **dCBP**. Energy gaps between the ground-state and the first triplet excited state for all the compounds examined here are larger than the 0.98 eV threshold that is the energy separation between the O_2_ triplet ground and the first singlet excited state. The influence of the presence of platinum is evident in the calculated values of the SOC elements. Indeed, all the calculated values for **mCBP** and especially for **dCBP** and their derivatives are significantly larger than those for free **BP**, and NTOs’ plots show a considerable metal participation in the triplet states.

## Supporting Information Available

Benchmark on the maximum absorption wavelength, λ_max_ in nm, for **BP** and **mCBP**; free energy profiles describing the first and second hydrolysis of **dCBP** together with the corresponding first and second guanine attacks to the aquated **dCBP** complex; relative energies in kcal mol^–1^ and calculated with respect to the first adduct formed in each described reaction; free energy profiles describing the first and second guanine attacks to the aquated simple pyrimidine-chelated cisplatin complex (**Pyrim**); excitation energies, absorption wavelength, oscillator strength, and MO contribution for Pt^II^ complexes and BODIPY; lowest triplet states excitation energies, absorption wavelength, oscillator strength, and MO contribution for Pt^II^ complexes and BODIPY; SOC values for the singlet–triplet radiationless transitions and relative energy gaps (eV) computed for all the investigated compounds; and highest occupied and lowest unoccupied natural transition orbitals (NTOs) of **BP**, **mCBP,** and its derivatives **mCBP**_**w**_ and **mCBP**_**g**_, and **dCBP** and its derivatives **dCBP**_**2w**_, **dCBP**_**wg**_, and **dCBP**_**2g**_ (PDF)
