# Supplementary material for: Computational Analysis of the Behavior of BODIPY Decorated Monofunctional Platinum(II) Complexes in the Dark and under Light Irradiation
Source: J Phys Chem A. 2022 Oct 4;126(40):7159–67. doi: 10.1021/acs.jpca.2c04544 (PMC9574924; doi:10.1021/acs.jpca.2c04544)
Supplement: Supplementary file 1 — jp2c04544_si_001.pdf [file jp2c04544_si_001.pdf]

Supporting Information

Computational Analysis of the Behaviour of BODIPY Decorated Monofunctional Pt(II) Complexes in Dark and under Light Irradiation

Pierraffaele Barretta,[a] Fortuna Ponte,[a] Stefano Scoditti,[a] Vincenzo Vigna,[a] Gloria Mazzone,[a*] Emilia Sicilia[a*]

[a]Department of Chemistry and Chemical Technologies, University of Calabria, Ponte P. Bucci, 87036 Arcavacata di Rende (CS), Italy.

Table of Contents

- **Table S1**: Benchmark on the maximum absorption wavelength, λ_{max} in nm, for **BP** and **mCBP** employing the 6-31G** basis set for C, N, H, Cl, B and F and the pseudopotential with its split-valence basis set SDD for Pt atom.
- **Figure S1**: Free energy profiles describing the a) first and b) second hydrolysis of **dCBP** together with the corresponding c) first and d) second guanine attacks to the aquated **dCBP** complex. Relative energies are in kcal mol^{-1} and calculated with respect to the first adduct formed in each described reaction.
- **Figure S2**: Free energy profiles describing the a) hydrolysis and b) guanine attack of cisplatin. Relative energies are in kcal mol^{-1} and calculated with respect to the first adduct formed in each described reaction.
- **Figure S3**: Free energy profiles describing the a) hydrolysis and b) guanine attack of **Pyr**. Relative energies are in kcal mol^{-1} and calculated with respect to the first adduct formed in each described reaction.
- **Figure S4**: Free energy profiles describing the a) first and b) second guanine attacks to the aquated simple pyrimidine-chelated cisplatin complex (**Pyrim**). Relative energies are in kcal mol^{-1} and calculated with respect to the first adduct formed in each described reaction.
- **Table S2**: Excitation energies (ΔE, eV), absorption wavelength (λ, nm), oscillator strength (*f*), MO contribution (%) for Pt^{II} complexes and BODIPY.
- **Table S3**: Lowest triplet states excitation energies (ΔE, eV), absorption wavelength (λ, nm), oscillator strength (f), MO contribution (%) for Pt^{II} complexes and BODIPY.
- **Table S4**: SOC values (cm^{-1}) for the $S_n \rightarrow T_m$ (with n = 1-4, m = 1-7) radiationless transitions and singlet–triplet energy gaps (eV) computed for all the investigated compounds.
- **Figure S5**: Highest occupied and lowest unoccupied natural transition orbitals (NTOs) of **BP**, **mCBP** and its derivatives **mCBP$_w$** and **mCBP$_g$**.
- **Figure S6**: Highest occupied (holes) and lowest unoccupied (particles) natural transition orbitals (NTOs) for singlet states of **dCBP** and its derivatives **dCBP$_{2w}$**, **dCBP$_{wg}$** and **dCBP$_{2g}$**.
- **Figure S7**: Highest occupied (holes) and lowest unoccupied (particles) natural transition orbitals (NTOs) for triplet states of **dCBP** and its derivatives **dCBP$_{2w}$**, **dCBP$_{wg}$** and **dCBP$_{2g}$**.

Table S1: Benchmark on the maximum absorption wavelength, λ_{max} in nm, together with the computed oscillator strength (*f*) for **BP** and **mCBP** employing the 6-31G** basis set for C, N, H, Cl, B and F and the pseudopotential with its split-valence basis set SDD for Pt atom. Data obtained with SCS-CC2 approach are also reported.

	**BP**		**mCBP**	
	λ_{max}	*f*	λ_{max}	*f*
Exp	*491*		*503*	
B3LYP	418	0.578	427	0.538
B3PW91	417	0.447	426	0.381
B97D	428	0.626	436	0.593
CAM-B3LYP	419	0.599	430	0.564
M06	423	0.564	433	0.558
M06L	411	0.643	418	0.538
MN12L	396	0.636	401	0.521
MN15L	393	0.415	400	0.333
PBE	429	0.596	437	0.558
PBE0	415	0.573	424	0.533
wB97X	423	0.651	434	0.558
SCS-CC2	431	0.558	441	0.621

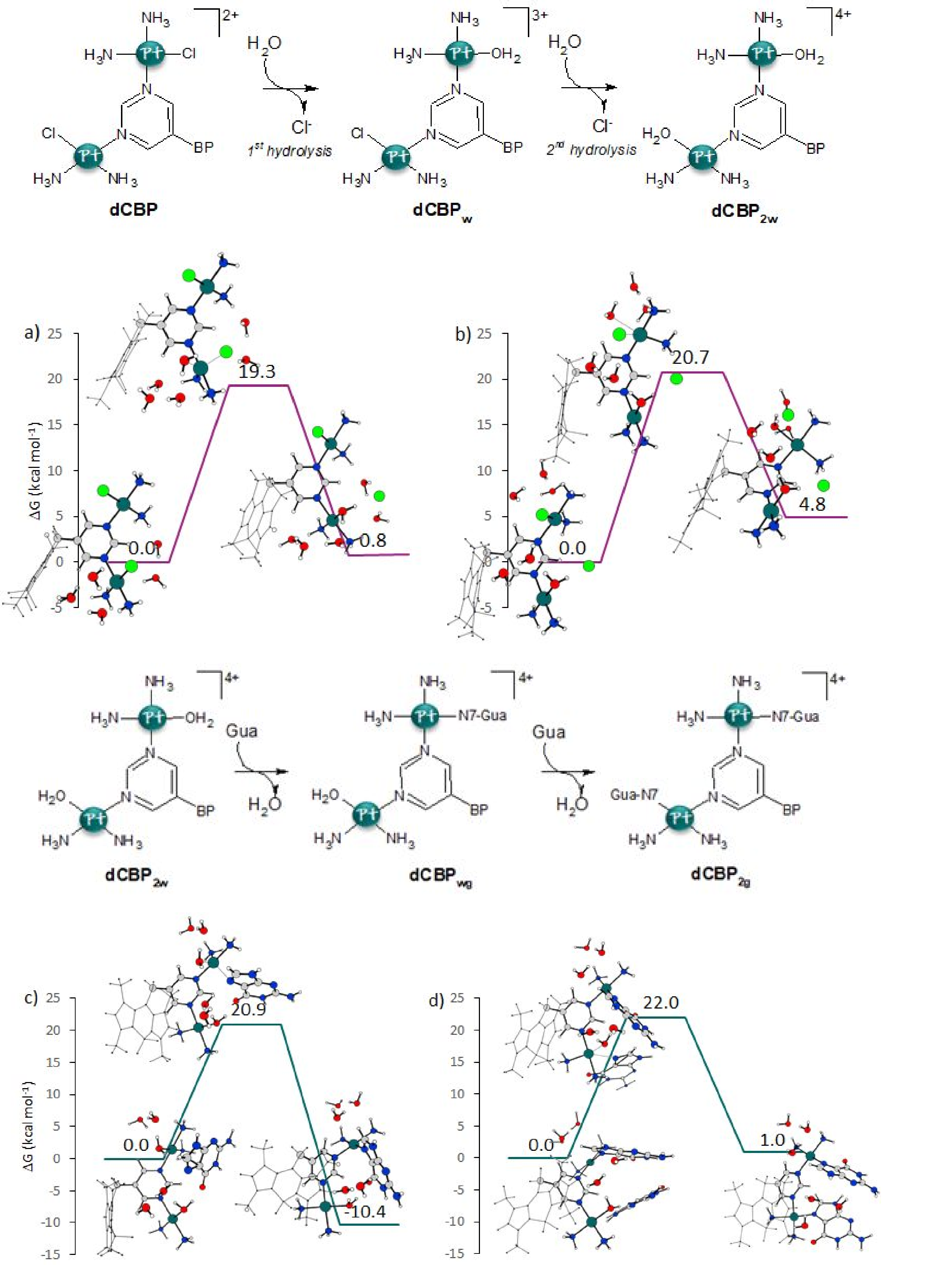

Figure S1: Free energy profiles describing the a) first and b) second hydrolysis of **dCBP** together with the corresponding c) first and d) second guanine attacks to the aquated **dCBP** complex. Relative energies are in kcal mol^{-1} and calculated with respect to the first adduct formed in each described reaction.

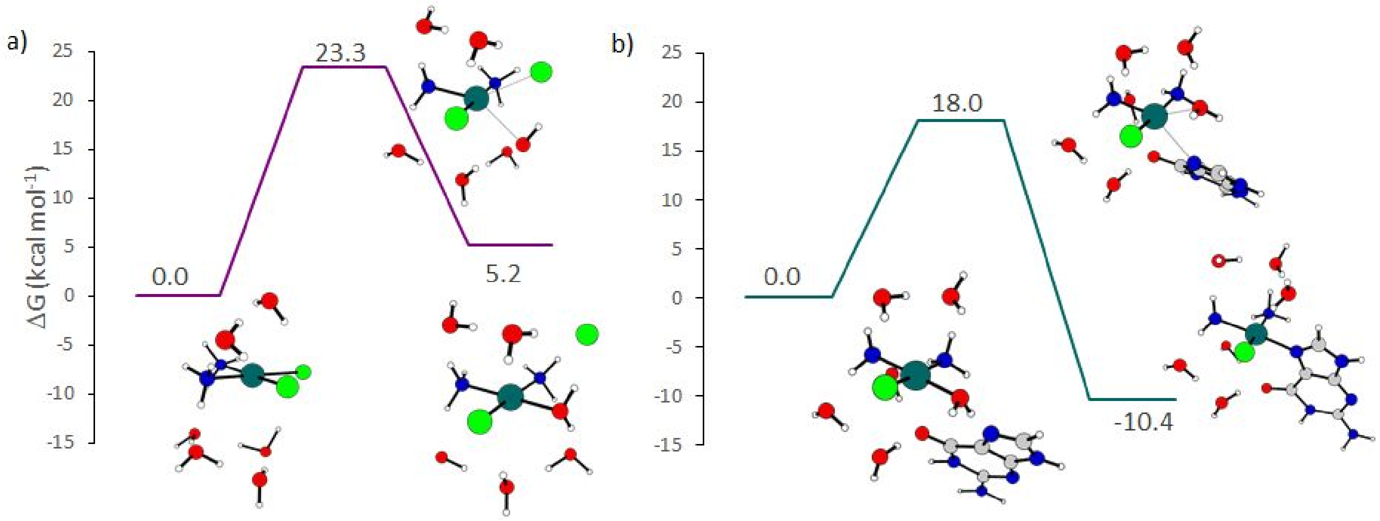

Figure S2: Free energy profiles describing the a) hydrolysis and b) guanine attack of cisplatin. Relative energies are in kcal mol^{-1} and calculated with respect to the first adduct formed in each described reaction.

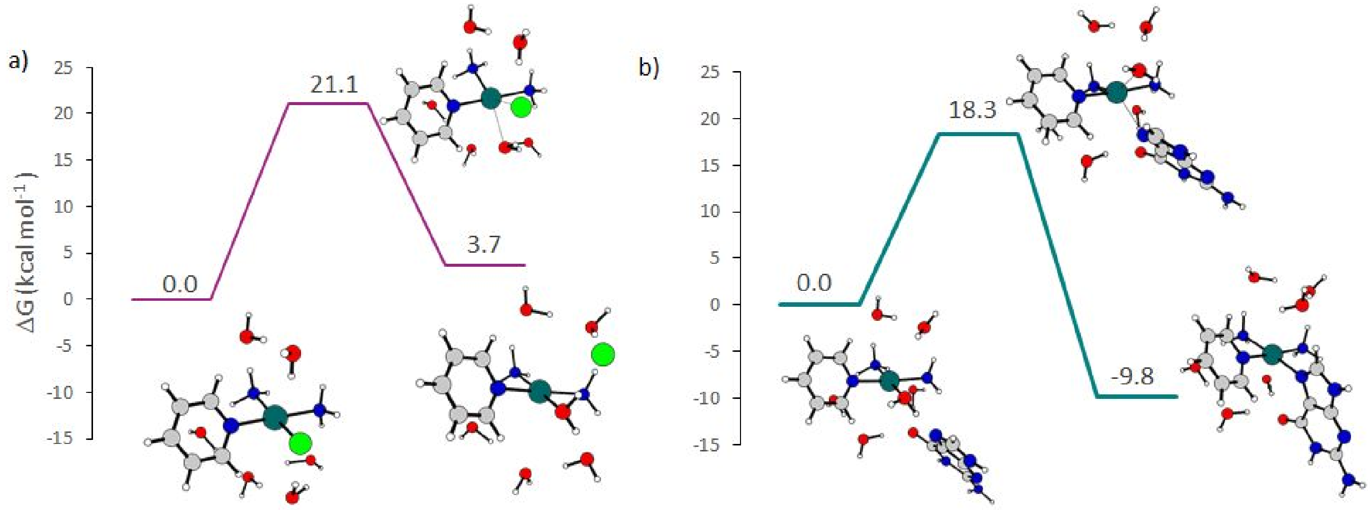

Figure S3: Free energy profiles describing the a) hydrolysis and b) guanine attack of **Pyr**. Relative energies are in kcal mol^{-1} and calculated with respect to the first adduct formed in each described reaction.

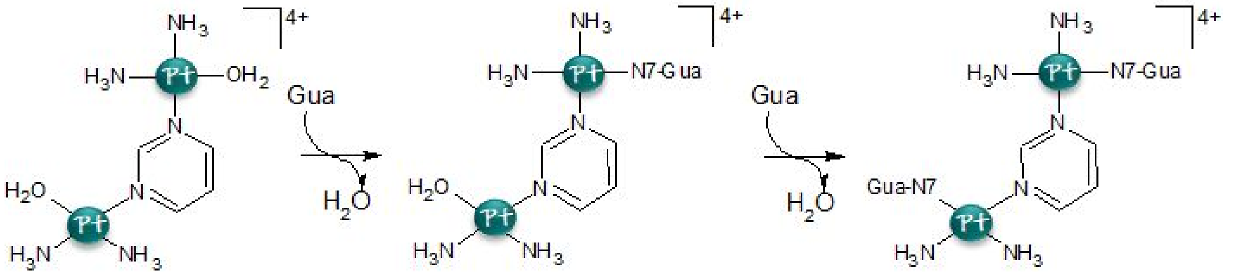

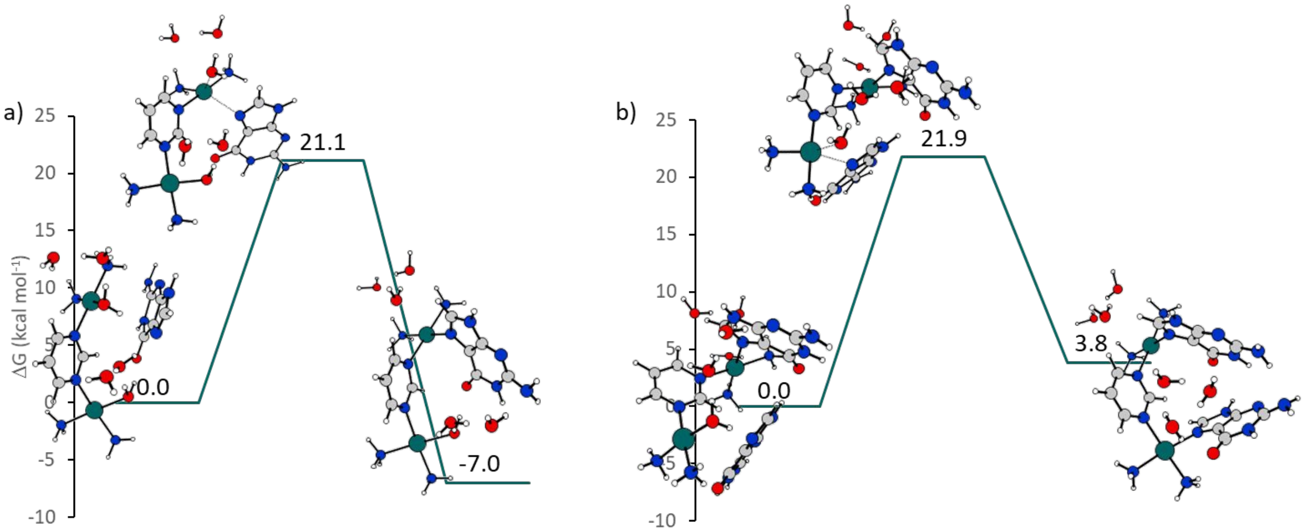

Figure S4: Free energy profiles describing the a) first and b) second guanine attacks to the aquated simple pyrimidine-chelated cisplatin complex (**Pyrim**). Relative energies are in kcal mol^{-1} and calculated with respect to the first adduct formed in each described reaction.

Table S2: Excitation energies (ΔE, eV), absorption wavelength (λ, nm), oscillator strength (f), MO contribution (%) for Pt^{II} complexes and BODIPY.

Compound	Band	ΔE	λ	f^a	MO contribution	Theoretical Assignment	$λ^{exp}$
**BP**	I	2.93	423	0.651	H → L 97%	ππ*	491[b]
	II	5.42	228	0.349	H-3 → L 94%	ππ*	
		7.76	160	0.254	H-2 → L+1 33%<br>H-1 → L+2 19%<br>H-9 → L 17%		
		8.10	153	0.210	H-9 → L 56%<br>H-1 → L+2 15%<br>H-11 → L 12%		
		8.17	152	0.403	H-1 → L+1 35%<br>H → L+3 31%<br>H-14 → L 13%		
		8.64	144	0.358	H-1 → L+3 40%<br>H-2 → L+2 36%		
**mCBP**	I	2.85	434	0.621	H → L 99%	ILCT	503[c]
	II	5.43	228	0.575	H-7 → L 52%<br>H-6 → L 34%	MLCT	
		6.08	204	0.204	H-5 → L+1 44%<br>H-9 → L+1 16%<br>H → L+4 10%		
**$mCBP_w$**	I	2.85	435	0.628	H → L 97%	ILCT	
	II	5.44	228	0.591	H-4→ L 90%	MLCT	
**$mCBP_g$**	I	2.85	435	0.616	H → L 97%	ILCT	
	II	5.15	241	0.177	H-1 → L+2 64%<br>H-1 → L+6 15%<br>H-1 → L+3 10%	MLCT	
		5.43	228	0.627	H-6 → L 78%		
**dCBP**	I	2.83	438	0.584	H → L 97%	ILCT	506[c]
	II	5.27	235	0.108	H-12 → L+1 27%<br>H-7 → L+1 14%<br>H-8 → L+1 11%<br>H-10 → L+1 11%	MLCT/ILCT	
		5.42	229	0.445	H-9 → L 73%		
**$dCBP_{2w}$**	I	2.79	444	0.564	H → L 97%	ILCT	
	II	5.28	235	0.414	H-5 → L 78%	MLCT	
		6.30	197	0.112	H-7 → L+2 31%<br>H-1 → L+2 21%<br>H → L+12 14%		
**$dCBP_{wg}$**	I	2.77	448	0.565	H → L 97%	ILCT	
	II	4.94	251	0.112	H-1 → L+4 38%<br>H-1 → L+1 32%	MLCT	
		5.25	236	0.288	H-6 → L 77%		
		5.80	215	0.232	H-1 → L+8 55%<br>H-1 → L+4 14%		
		6.65	186	0.119	H-4 → L 27%<br>H-8 → L+4 19%		
**$dCBP_{2g}$**	I	2.11	588	0.016	H → L 100%	LMCT	
		2.72	456	0.009	H → L+2 98%	LMCT	
		2.84	437	0.000	H-1 → L 100%	M_1LCT/LM_2CT[d]	
		2.87	433	0.504	H → L+1 96%	ILCT	
	II	5.09	244	0.123	H-1 → L+8 53%<br>H-1 → L+9 23%<br>H-1 → L+7 11%	ML_gCT/L_gL_gCT[e]	
		5.20	239	0.251	H-2 → L+8 58%<br>H-1 → L+9 14%		

a. only vertical transitions with oscillator strength greater than 0.1 are reported, with the exception of **$dCBP_{2g}$** for which all the singlet states lying below the bright one are reported in grey; b. from *Dyes and Pigments* 116 (2015) 74-81 in acetonitrile; c. from *Inorg. Chem.* 2021, 60, 10047−10055; d. subscripts 1 and 2 stand for platinum 1 and 2; e. the ligand L is in all cases the guanine base coordinated to the platinum.

Table S3: Lowest triplet states excitation energies (ΔE, eV), absorption wavelength (λ, nm), oscillator strength (f), MO contribution (%) for Pt^{II} complexes and BODIPY.

Compound	State	ΔE	MO contribution	Theoretical Assignment
**BP**	T1	1.27	H → L 100%	ππ*
**mCBP**	T1	1.08	H → L 100%	ILCT
	T2	2.61	H-2 → L+2 88%	MC/MLCT
	T3	2.82	H-3 → L+2 78%	MC/MLCT
			H-10 → L+2 12%	
**mCBP$_w$**	T1	1.06	H → L 100%	ILCT
	T2	2.42	H-3→ L+2 81%	MC/MLCT
			H-3→ L+3 11%	
**mCBP$_g$**	T1	1.07	H → L 100%	ILCT
**dCBP**	T1	1.05	H → L 100%	ILCT
	T2	2.47	H-3 → L+4 39%	MLCT/MC
			H-3 → L+3 35%	
			H-2 → L+3 16%	
	T3	2.48	H-2 → L+4 42%	MLCT/MC
			H-2 → L+3 30%	
			H-3 → L+3 16%	
	T4	2.71	H-6 → L+3 45%	MLCT/MC
			H-6 → L+4 36%	
	T5	2.72	H-5 → L+4 40%	MLCT/MC
			H-5 → L+3 39%	
**dCBP$_{2w}$**	T1	1.02	H → L 100%	ILCT
	T2	2.25	H-4 → L+3 64%	MLCT/MC
			H-4 → L+4 28%	
	T3	2.30	H-3 → L+4 58%	MLCT/MC
			H-3 → L+3 30%	
**dCBP$_{wg}$**	T1	1.00	H → L 100%	ILCT
	T2	2.33	H-5 → L+3 71%	MLCT/MC
			H-3 → L+3 10%	
	T3	2.71	H-4 → L+5 66%	MLCT/MC
			H-3 → L+5 10%	
**dCBP$_{2g}$**	T1	1.46	H → L+1 100%	ILCT
	T2	2.08	H → L 100%	LMCT/LLCT
	T3	2.66	H-3 → L+1 95%	ILCT
	T4	2.71	H → L+2 99%	LLCT
	T5	2.82	H-1 → L 99%	LLCT
	T6	2.84	H-5 → L+1 78%	ILCT
	T7	2.86	H-6 → L+3 61%	MC/MLCT
			H-6 → L+4 13%	

Table S4: SOC values (cm^{-1}) for the $S_n \rightarrow T_m$ (with n = 1-4, m = 1-7) radiationless transitions and singlet–triplet energy gaps (eV) computed for all the investigated compounds.

	**BP**		**mCBP**		**$mCBP_w$**		**$mCBP_g$**	
n,m	SOC	ΔE	SOC	ΔE	SOC	ΔE	SOC	ΔE
1,1	0.05	1.66	0.03	1.77	0.07	1.79	0.02	1.58
1,2			2.54	0.24	0.10	0.43		
1,3			0.72	0.03				
	**dCBP**		**$dCBP_{2w}$**		**$dCBP_{wg}$**		**$dCBP_{2g}$**	
1,1	0.18	1.78	0.22	1.77	0.45	1.77	0.37	0.65
1,2	22.44	0.36	1.22	0.54	8.23	0.44	0.21	0.03
1,3	3.95	0.35	2.48	0.49	0.41	0.06		
1,4	0.73	0.12						
1,5	4.86	0.11						
2,1							4.85	1.26
2,2							0.52	0.64
2,3							0.47	0.06
2,4							17.64	0.01
3,1							1.24	1.38
3,2							0.94	0.76
3,3							8.43	0.18
3,4							37.10	0.13
3,5							28.43	0.02
3,6							74.20	0.00
4,1							1.73	1.41
4,2							0.46	0.79
4,3							2.51	0.21
4,4							26.90	0.16
4,5							24.46	0.05
4,6							90.78	0.03
4,7							830.11	0.03

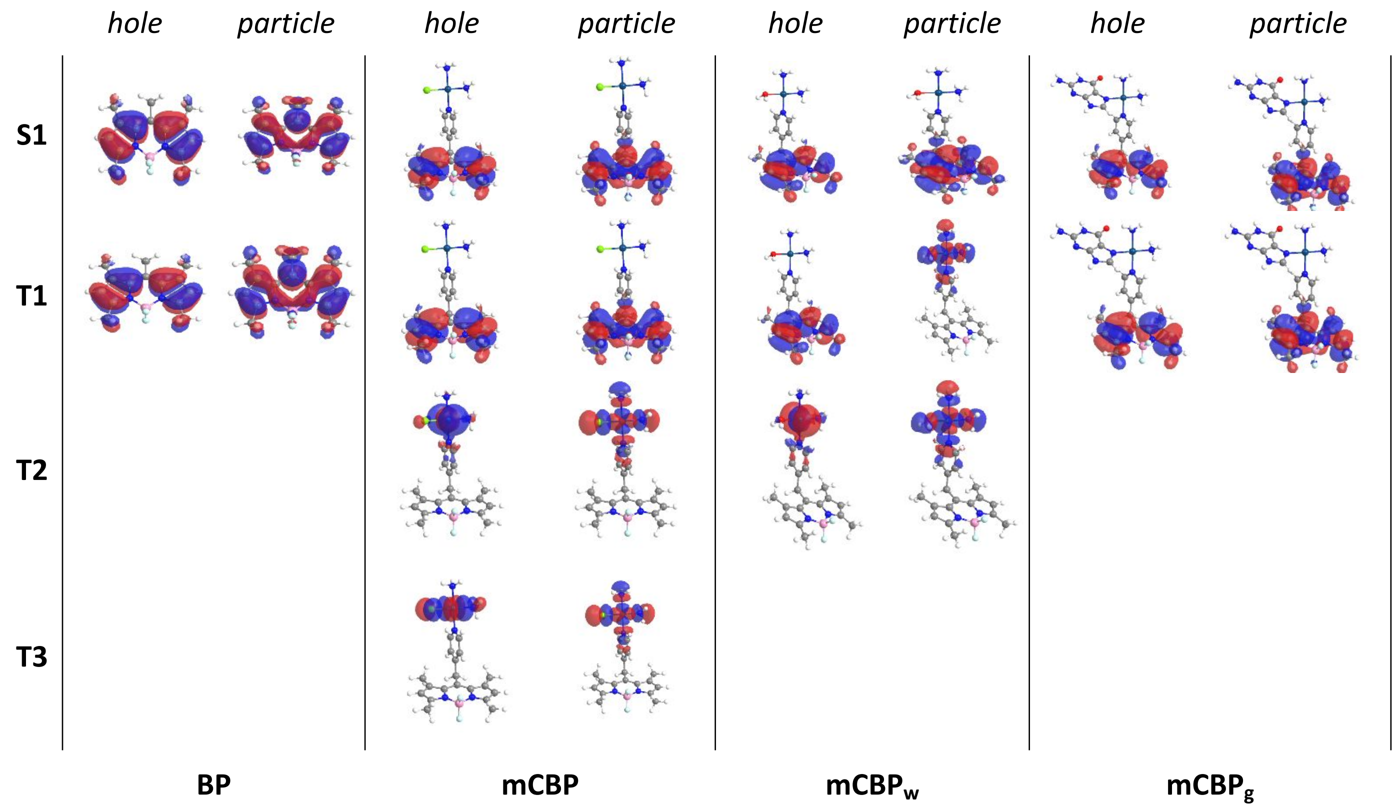

Figure S5: Highest occupied and lowest unoccupied natural transition orbitals (NTOs) of **BP**, **mCBP** and its derivatives **mCBP$_w$** and **mCBP$_g$**.

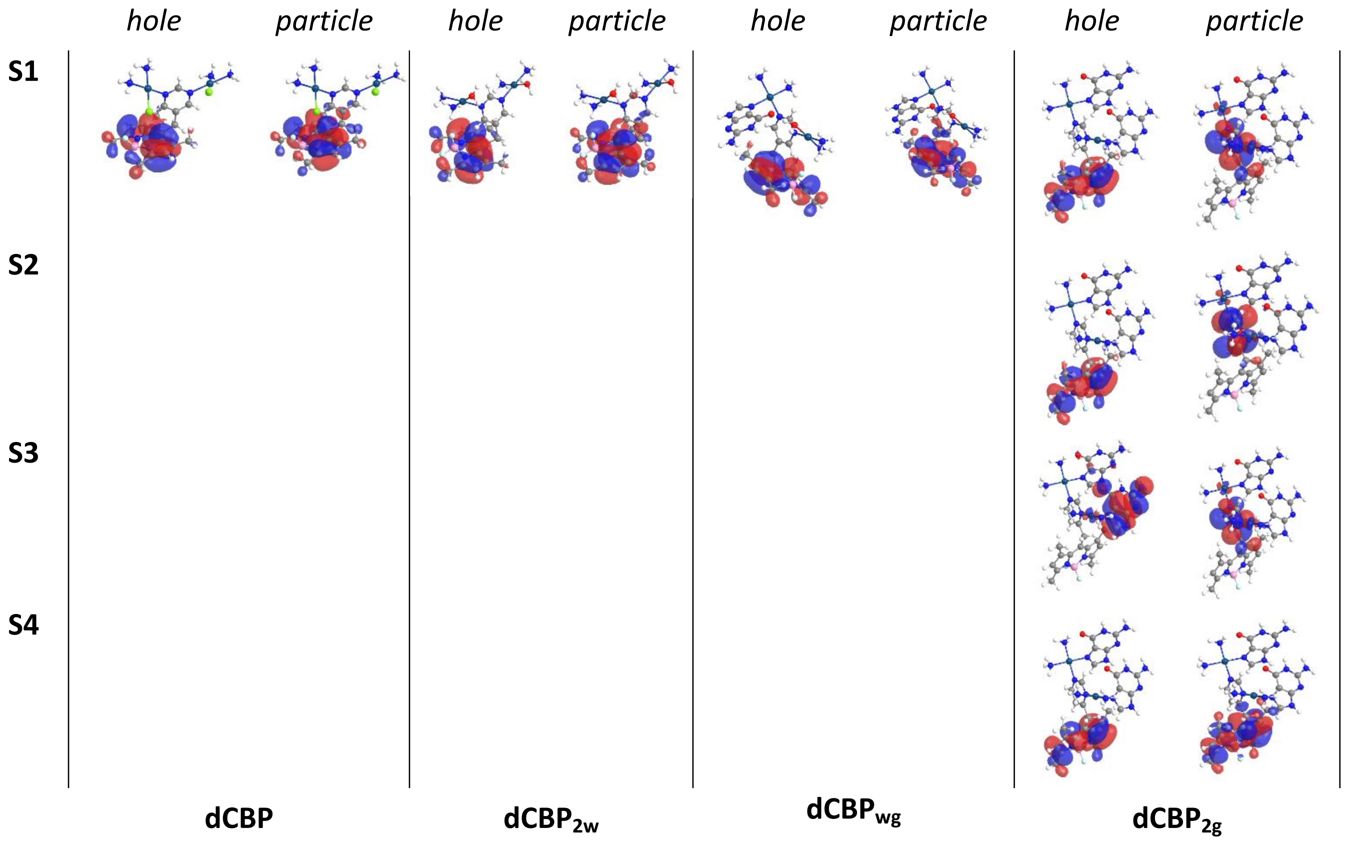

Figure S6: Highest occupied (holes) and lowest unoccupied (particles) natural transition orbitals (NTOs) for singlet states of **dCBP** and its derivatives **dCBP$_{2w}$**, **dCBP$_{wg}$** and **dCBP$_{2g}$**.

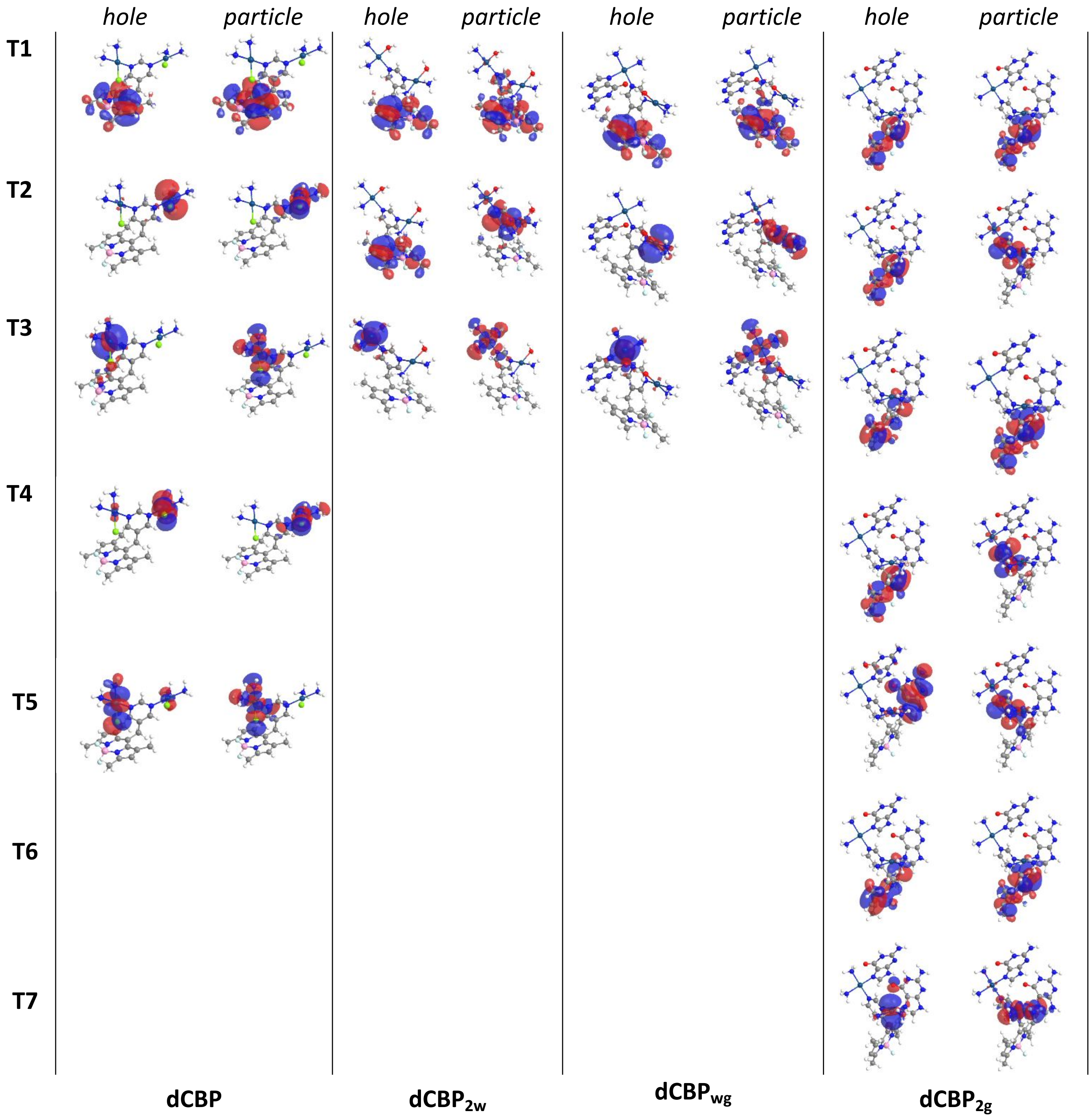

Figure S7: Highest occupied (holes) and lowest unoccupied (particles) natural transition orbitals (NTOs) for triplet states of **dCBP** and its derivatives **dCBP$_{2w}$**, **dCBP$_{wg}$** and **dCBP$_{2g}$**.
